# *Neuronal Signaling*: an introduction

**DOI:** 10.1042/NS20160025

**Published:** 2016-10-18

**Authors:** Aideen M. Sullivan

**Affiliations:** Editor-in-Chief, *Neuronal Signaling*, and Department of Anatomy and Neuroscience, University College Cork, Cork, Ireland

## Abstract

There have been a number of advances in our knowledge of neuronal communication in processes involved in development, functioning and disorders of the nervous system. This progress has prompted the Biochemical Society to launch *Neuronal Signaling*, a new open access journal that aims to expand on the existing knowledge about signaling within and between neurons.

Communication between and within neurons is critical for all functions of the nervous system, from development to aging, through health and disease. The last decade has seen huge advances in our knowledge of the molecular, cellular and systematic signaling pathways within the nervous system. There have been significant breakthroughs in studies on the signaling pathways that underlie neurogenesis [1], addiction [2,3] and autism spectrum disorders [4,5], as well as the pathophysiology and treatment of mood disorders [6,7].

Technological advances such as the development and application of optogenetics [8,9], and remarkable achievements in genetic reprogramming of adult cells [10], have given us powerful tools to study and manipulate neurons in the laboratory. Optogenetics has transformed neuroscience by allowing precise spatiotemporal manipulation of individual neuronal cells and circuits [11,12], enabling significant insights in the areas of neurophysiology, behavioral and cognitive neuroscience, and neuropsychiatric disease, among others.

The remarkable discovery in 2006 of induced pluripotent stem cells (iPSCs) [13] showed us that a mature cell can be manipulated in culture to become a completely different cell type, even a highly-differentiated functional neuron. This has revolutionized the field of regenerative medicine and has enabled precise modelling of diseases in the laboratory [14]. The discovery of iPSCs has huge implications for neuroscience as it allows the study of pathogenic signaling mechanisms involved in neurological diseases, as well as the development of new therapeutic approaches [15].

Advances in our knowledge of epigenetic mechanisms, and how they shape the developing and aging nervous system [16–18], have contributed much to the study of complex neurodegenerative and psychiatric disorders, and will be critical for driving progress in their diagnosis and targeted treatment.

The large extent and vast scope of these advances in our knowledge of neuronal communication invites the creation of a new journal specializing in research on the molecular and signaling processes involved in the development and functioning of the nervous system, as well as its disorders. Furthermore, crossover between disciplines in biological research is central to driving the advancement of our collective knowledge, and breakthroughs in neurological therapeutics and patient care have often been fueled by findings in other disciplines. For example, much of the information on the signaling pathways used by neurons has been gleaned from the fields of oncology and developmental biology [19–23].

The Biochemical Society's mission to advance the molecular biosciences as a whole is not restricted by specialty and the Society was therefore keen to launch a new journal exploring molecular biology of the brain, and signaling networks within and between neurons. Having accepted the Editor-in-Chief position for this new journal, I am now delighted and honored to introduce *Neuronal Signaling*, a new, online-only, fully open access (OA) journal, published by Portland Press, the Society's wholly-owned trading subsidiary. This journal will be the first fully OA journal focusing on signaling aspects of neuroscience to be owned by a European society and will aspire to apply the expertise of the Biochemical Society in cellular and molecular signaling to the field of neuroscience. It will disseminate peer-reviewed articles at the interface of neuroscience and molecular biology, with original research articles and reviews on all aspects of inter- and intra-neuronal signaling.

*Neuronal Signaling* will span a large range of molecular and cellular neuroscience research topics, from the development of the nervous system through to its aging, from its normal functioning to a range of neurological and neuropsychiatric disorders. The journal invites the submission of excellent original research articles, as well as review articles on pertinent topics in any aspect of neuronal signaling. We will ensure that all manuscripts are peer-reviewed in a fair and timely manner.

All *Neuronal Signaling* articles will be published as OA under the liberal Creative Commons CC BY license ensuring compliance with current research-funder OA policies. This OA policy is in keeping with the recent trend of ever-increasing numbers of research articles in fully-OA journals being published under the CC BY license (see http://oaspa.org/oaspa-members-ccby-growth-2015-data/). All researchers publishing in *Neuronal Signaling* will therefore benefit from maximal visibility and dissemination of their published work.

The Biochemical Society's mission is to promote the future of molecular biosciences by facilitating the sharing of expertise, supporting the advancement of biochemistry and molecular biology, and raising awareness of their importance in addressing challenges within society. Portland Press as the Society's wholly-owned trading subsidiary, is embedded in the global scientific community and dedicated to promoting and sharing scientific research. All surpluses from Portland Press’ trading activities are gifted back to the Society, to support the advancement of the molecular life sciences through activities such as scientific meetings, public engagement and training events, awards, support for educators, students and early-career researchers, and the provision of grants and bursaries.

Both Portland Press and I recognize the growing need to link OA science, which is by design available for *all* to read, with what the findings from a published article might mean for an individual (including patients and their relatives) and for society in general. We therefore intend to conclude articles with summaries that point to what the research findings or review-article conclusions might mean for a patient or caregiver. Not only will this make important scientific findings reported in the journal accessible to any reader, but, will also help authors of published articles report on the human and societal impacts of their findings.

I am very much looking forward to working on this journal with our international team of expert Associate Editors, chosen from across the neurosciences, which includes Eero Castrén (University of Helsinki, Finland), Eilís Dowd (NUI Galway, Ireland), Meng Li (Cardiff University, UK), Clare Stanford (University College London, UK) and Vidita Vaidya (Tata Institute of Fundamental Research, Mumbai, India).
